# Gender and Intersecting Barriers and Facilitators to Access the HIV Cascade of Care in Manitoba, Canada, Before and During the COVID-19 Pandemic: A Qualitative Study

**DOI:** 10.3390/tropicalmed9120287

**Published:** 2024-11-25

**Authors:** Enrique Villacis-Alvarez, Cheryl Sobie, Katharina Maier, Margaret Lavallee, Chantal Daniels, Heather Pashe, Joel Baliddawa, Nikki Daniels, Rebecca Murdock, Robert Russell, Clara Dan, Freda Woodhouse, Susie Cusson, Lisa Patrick, Marj Schenkels, Michael Payne, Ken Kasper, Lauren J. MacKenzie, Laurie Ireland, Kimberly Templeton, Kathleen Deering, Margaret Haworth-Brockman, Yoav Keynan, Zulma Vanessa Rueda

**Affiliations:** 1 Department of Medical Microbiology and Infectious Diseases, Rady Faculty of Health Sciences, University of Manitoba, Winnipeg, MB R3E 0J9, Canada; villacisenrique@gmail.com (E.V.-A.); yoav.keynan@umanitoba.ca (Y.K.); 2 Criminal Justice, The University of Winnipeg, Winnipeg, MB R3B 2E9, Canada; k.maier@uwinnipeg.ca; 3 Ongomiizwin Indigenous Institute of Health & Healing, Rady Faculty of Health Sciences, University of Manitoba, Winnipeg, MB R3E 0W2, Canada; 4 Peer Research Team, Alltogether4IDEAS, Winnipeg, MB R3E 0J9, Canada; 5 Nine Circles Community Health Centre, Winnipeg, MB R3G 0X2, Canada; 6 The Manitoba HIV Program, Winnipeg, MB R3G 0X2, Canada; 7 Department of Internal Medicine, Rady Faculty of Health Sciences, University of Manitoba, Winnipeg, MB R3E 0J9, Canada; 8 Department of Family Medicine, Rady Faculty of Health Sciences, University of Manitoba, Winnipeg, MB R3E 0J9, Canada; 9 Division of Medicine, Department of Medicine, Faculty of Medicine, University of British Columbia, Vancouver, BC V6Z 2K5, Canada; kathleen.deering@cgshe.ubc.ca; 10 Centre for Gender and Sexual Health Equity, Faculty of Medicine, University of British Columbia, Vancouver, BC V6Z 2K5, Canada; 11 Department of Community Health Sciences, Rady Faculty of Health Sciences, University of Manitoba, Winnipeg, MB R3E 0J9, Canada; margaret.haworth-brockman@umanitoba.ca; 12 National Collaborating Centre for Infectious Diseases, Rady Faculty of Health Sciences, University of Manitoba, Winnipeg, MB R3E 0J9, Canada; 13 Escuela de Ciencias de la Salud, Universidad Pontificia Bolivariana, Medellin 050021, Colombia

**Keywords:** HIV, syndemic, barriers to HIV care, person-centered care, qualitative research, community-based research

## Abstract

Marginalized groups in Manitoba, Canada, especially females and people who inject drugs, are overrepresented in new HIV diagnoses and disproportionately affected by HIV and structural disadvantages. Informed by syndemic theory, our aim was to understand people living with HIV’s (PLHIV) gendered and intersecting barriers and facilitators across the cascade of HIV care before and during the COVID-19 pandemic. This study was co-designed and co-led alongside people with lived experience and a research advisory committee. We employed semi-structured interviews with thirty-two participants and three questionnaires. Interviews were audio-recorded, transcribed, and coded, and descriptive statistics were performed on the first two questionnaires. Qualitative data analysis used thematic analysis and focused on identifying categories (individual, healthcare, and social/structural) related to the barriers and facilitators to HIV care. A total of 32 PLHIV completed this study and over 70% of females and 50% of males reported severe and moderate sexual abuse among other traumatic childhood experiences. Barriers to accessing or continuing in the cascade of HIV care included navigating the initial shock of receiving an HIV diagnosis, mental health challenges and inaccessible supports, substance use, violence (including intimate partner), internalized and enacted compounded stigma related to houselessness and substance use, discrimination by primary care service providers and social networks, lack of preventative and social supports, lack of accessible housing, and programmatic issues. COVID-19 increased mental health problems and disrupted relationships with HIV service providers and peers living with HIV. Facilitators to HIV care included stopping substance use, caring service providers particularly during HIV diagnosis, welcoming healthcare environments, social opportunities and integrated supports, and supportive social networks. Women, men, and non-binary PLHIV experience interconnected factors complicating their experiences with HIV care. Interventions should consider holistic, person-centered, and trauma-informed care options to address the barriers found in this research and appropriately serve PLHIV.

## 1. Background

The Joint United Nations Programme on HIV/AIDS estimated that approximately 39 million people worldwide were living with HIV in 2022 [1]. In 2022, the Government of Canada reported a decrease in HIV diagnoses and achievement of the first and third objectives of the 90-90-90 goals [2]. However, the landscape of HIV in Canada is heterogeneous.

The Canadian province of Manitoba reported a 52% increase in new HIV diagnoses between 2018 and 2021, with increased diagnoses among females, those who self-identify as Indigenous, people who engage in heterosexual sex, and those who inject methamphetamines, and an increase in people not returning to HIV care [3,4,5]. Epidemiological data from new HIV diagnoses reported a disease cluster of increased concurrent sexually transmitted and blood-borne infections (STBBIs) and mental health conditions combined with houselessness (Supplementary File S1) [4,5]. In addition, the COVID-19 pandemic exacerbated health disparities among people living with HIV (PLHIV) [6]. Indigenous people account for 18% of the population of Manitoba [7], yet they are overrepresented in new HIV diagnoses (76% from 2018 to 2021) [4,5], houselessness counts [8], and other health conditions which stem from the past and current harms of colonization and violent structural policies [9].

The biological interactions of the HIV and STBBI disease cluster may lead to worse health outcomes by increasing the transmission of each or exacerbating disease progression. For example, inflammatory immune cell responses induced by STBBIs can increase the risk of HIV transmission [10]. The mutually reinforcing biological implications of HIV and substance use have also been well documented [11,12,13] and, in particular, methamphetamine use has been associated with a higher rate of HIV viral replication [14]. Preclinical data demonstrate that the use of injection drugs and the presence of STBBIs are powerful independent cofactors that disrupt tissues, favor inflammation, and promote HIV acquisition and poor health outcomes. Specifically, methamphetamine use is associated with changes to mucosal tissues, which are linked to inflammation and immune activation [15,16,17]. Genital inflammation significantly increases the risk of HIV acquisition due to the recruitment of HIV target cells (activated T cells) to the genital tract. Activated CD4+ T cells are more susceptible to HIV infection than quiescent cells and once infected, produce up to 1000 times more virus [18]. In addition, the recent use of methamphetamine among PLHIV affected T cell function [19,20] and was associated with inflammation and vascular injury [21].

In addition, psychosocial factors such as mental health disorders and trauma, houselessness, marginalization, and stigma and discrimination can negatively impact health outcomes of PLHIV. People experiencing mental health disorders have been overrepresented in HIV diagnoses [22]. The social and structural factors associated with homelessness have been shown to result in altered immune function and high levels of oxidative stress along with impaired NK and interleukin responses [23]. It is plausible that the social, substance use-related, and STBBI-induced immune dysregulation act in concert to increase the risk of HIV acquisition. In addition, researchers have found that people experiencing more severe mental health disorders, homelessness, and blood-borne infections tend to utilize primary and specialized health services at lower rates compared to those with less severe mental health disorders and comorbidities [24]. Qualitative studies have highlighted that substance use and active mental health disorders may increase the risk of apathy about care for PLHIV, resulting in missed appointments and reduced medication adherence [25]. Similarly, stigma and discrimination towards PLHIV are linked to lower retention in HIV care and lower overall healthcare utilization regardless of gender [26,27].

There are numerous facilitators and barriers across the continuum of HIV care. While some affect all sub-groups of PLHIV in Canada [28], others are more specific to contexts and populations. For instance, Gahagan and colleagues documented gender-based testing barriers related to gendered expectations and roles among heterosexual men in Nova Scotia [29]. Barriers for street-involved youth in Canada included intersectional stigma, discrimination, and lack of support for basic needs [30], while affordable and wrap-around services worked as facilitators. In an Ontario-based study, barriers for racial and ethnic minority middle-aged and older men who have sex with men included language, racism, cultural norms, and stigma, while wrap-around community-based services promoting HIV resilience and care engagement enabled their HIV care [31].

Understanding that co-occurring epidemics interact with each other and are shaped by context-specific social factors, our study is informed by syndemic theory originally proposed by Singer [11,32]. The syndemic framework proposes the existence of a cluster of distinct yet interconnected diseases which influence each other at different levels (e.g., biologically, socially, and/or psychologically) within specific social forces that reinforce and maintain these clusters [11,33,34]. Syndemic theory goes further than simply documenting the existence of comorbid diseases as it recognizes that social realities mold the presence of and interactions between diseases which can posit adverse interactions among underserved populations [35]. The present article acknowledges the current cluster of diseases and psychosocial conditions experienced by PLHIV in Manitoba [4,5] and aims to disentangle the gendered and intersecting barriers and facilitators to accessing and remaining in the HIV cascade of care. Our major emphasis is on describing the biological and social interactions that are shaping PLHIV’s HIV care utilization to inform public health and clinical interventions across the province.

## 2. Materials and Methods

### 2.1. Ethics

The protocol for this research has been published elsewhere [32]. This study was approved by the University of Manitoba Health Ethics Research Board (HS25572; H2022:218), the First Nations Health and Social Secretariat of Manitoba, Nine Circles Community Health Centre, Shared Health Manitoba (SH2022:194), and the 7th Street Health Access Centre. 

### 2.2. Study Setting

This study took place in the province of Manitoba, located in central Canada, which has a population of approximately 1.4 million; the majority of people live in Winnipeg (~900,000) and Brandon (~55,000) [36]. The rest of the population is spread across many smaller towns and communities. Anyone diagnosed with HIV in Manitoba is referred to the centralized Manitoba HIV program. Specialized HIV care is given at three clinics across the province, two located in Winnipeg and one in Brandon. Data were collected at the three Manitoba HIV program sites. At the time of data collection, people with a provincial health card received their specialized HIV care as part of the province’s insured services. However, the costs of medications depended on individual situations. For example, people employed with benefits may have had their prescriptions paid for by their employers’ health plans. Other employed people may have had their medication costs covered through a provincial insurance plan which requires a deductible each year, depending on annual income.

### 2.3. Researchers Reflexivity

This study is situated within a larger collaborative multidisciplinary project bringing together academic researchers, people with lived experiences, community-based researchers, and medical and social service providers across Canada. The Alltogether4IDEAS research team came together after initial meetings between the three principal investigators and knowledge users (e.g., HIV physicians and health care personnel, and people living with HIV).

**Figure 1:**
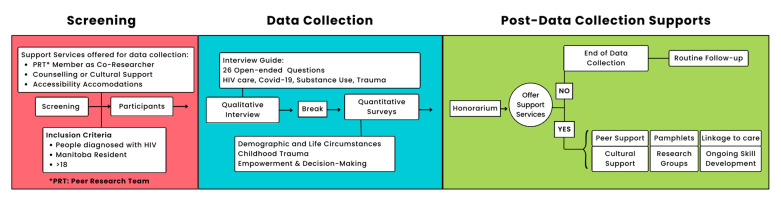
Data collection process. Our approach to inviting people living with HIV to participate in the research project is grounded in cultural safety, trauma-informed care, and harm reduction.

Guidance from the project’s research advisory committee, peer research team, cultural advisor, and community members resulted in a recruitment strategy grounded in trauma-informed principles, harm reduction, and cultural safety [32]. Participant engagement reflected a non-judgmental and non-stigmatizing approach, and we offered culturally appropriate supports for Indigenous participants [32] (Figure 1). People with lived experience co-designed and co-authored this research, provided extensive reviews and edits on data collection tools and procedures, participated in the analysis and interpretation of results, and reviewed, edited, and approved this article [32].

### 2.4. Participants

The inclusion criteria were PLHIV of 18+ who resided in Manitoba. We used purposive sampling to yield a diverse sample of men, women, and non-binary persons, as well as people from various race/ethnicity backgrounds. Our sampling strategy was carefully designed to hear stories from people diagnosed with HIV. We emphasized including people who have had smooth journeys in connecting and maintaining HIV care, and others who faced more barriers across their HIV care. We wanted to hear stories from people who are representative of the disease clusters recently reported among those with new HIV diagnoses in Manitoba, including experiences of houselessness and substance use [4,5]. The recruitment process included referrals from HIV service providers, posters and flyers in HIV and community organizations, social media, and in-person outreach at Manitoba HIV program sites. We stopped sampling once thematic saturation was reached.

### 2.5. Data Collection

Data were collected by the research associate (RA), peer co-researchers, and the Indigenous cultural advisor between October 2022, and May 2023. All data collection took place in a private room within at HIV clinics. Participants gave written or verbal informed consent at the start of a session, and then answered questions in an in-depth semi-structured interview inquiring about journey of HIV care, recommendations to HIV care, COVID-19, substance use, harm reduction services, and experiences of trauma. The interview guide was co-developed with people with lived experiences and was published in this study’s protocol [32]. After a break, participants responded to the following three survey instruments: a Sociodemographic and Life Circumstances Questionnaire (Supplementary File S2), a Childhood Trauma Questionnaire [37], and an Empower-Making Decisions Survey [38]. The Sociodemographic and Life Circumstances Questionnaire was co-developed through in-depth consultations with people with lived experiences, academic researchers, and HIV knowledge users. Specific questions and goals for understanding key factors related to participants’ health and social positions can be found in this study’s protocol [32]. All participants had the chance of completing the questionnaires themselves or have the research associate assist with completion orally. All questionnaires were paper-based, and the data were entered in a password-protected database. Data collection sessions took between one and two and a half hours. Interviews were audio recorded, and then transcribed using Otter.ai and reviewed for accuracy by the RA. This qualitative research was part of a broader mixed-methods study; therefore, participant sociodemographic information and responses from the Childhood Trauma Questionnaire are presented below to complement our qualitative findings. The results from the Empower-Making Decisions Survey will be presented in a future paper.

### 2.6. Data Analysis

**Figure 2:**
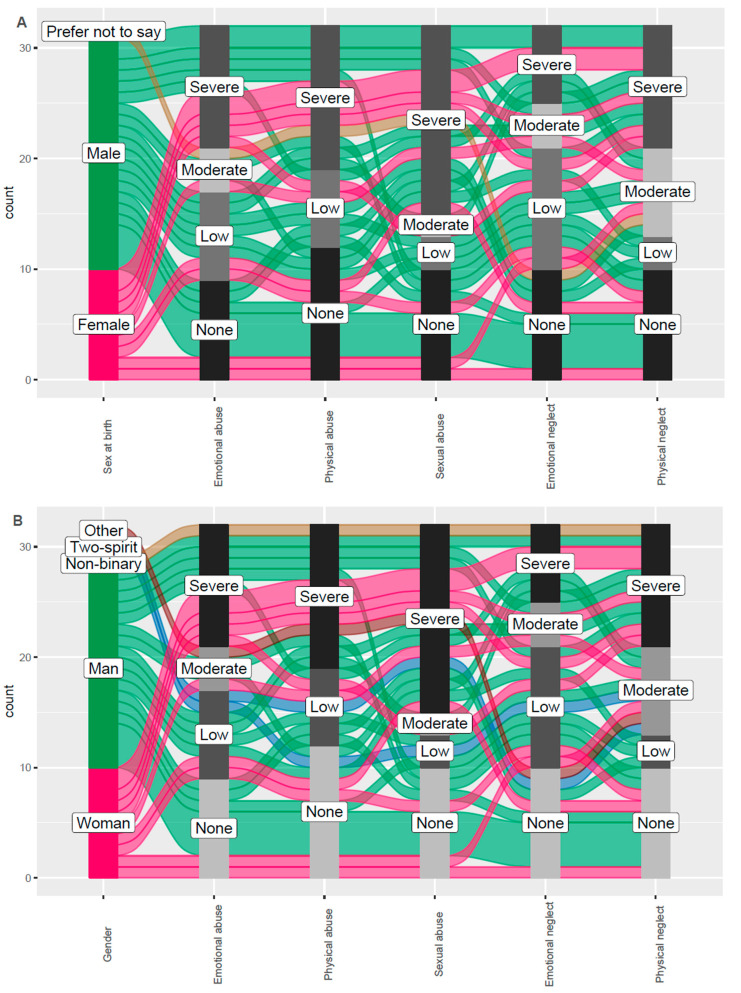
Severity of trauma for each of type of childhood trauma experienced by people living with HIV in Manitoba by sex (**A**) and gender (**B**). Severity score of trauma: none, low, moderate, and severe. Type of childhood trauma: emotional, physical and sexual abuse, and emotional and physical neglect.

Descriptive statistics were calculated for participant demographics. The scoring key for the Childhood Trauma Questionnaire was used to illustrate percentages of severity (e.g., none, low, moderate, severe) across five types of childhood trauma (i.e., emotional abuse, physical abuse, sexual abuse, emotional neglect, physical neglect). Childhood trauma was depicted by sex and gender, and Figure 2 was created using Jamovi^®^ software version 2.5. Along with the syndemic theory described above, we used the World Health Organization’s Conceptual Framework for Action on the Social Determinants of Health [32,39] as a theoretical approach to differentiate between the barriers and facilitators at three levels of individual, healthcare, and social and structural. This framework enables researchers to discern the social, economic, and policies that contextualize socioeconomic positions influencing people’s opportunities in society [39].

We used NVivo^®^ 12 Pro to thematically analyze the qualitative data. We employed thematic analysis, an approach to analyzing qualitative data originally developed by Braun and Clarke [40]. Since its inception, the methodology has gone through several revisions by its authors, yet this method enables researchers to recognize their own positionality and subjectivity to identify patterns and themes in the qualitative data [41]. The data analysis was led with the open coding of five transcripts by EVA who collected the data and spent extended periods of time in HIV clinics and field sites. CS, ZR, KM, and YK, who are trained researchers with experience in HIV research, reviewed the open coding and collaboratively discussed and compared codes to agree on a framework that would capture barriers and facilitators affecting PLHIV’s engagement with HIV care. A framework was developed to code barriers and facilitators at the three levels of individual, healthcare, and social and structural levels. Next, EVA continued coding the remaining transcripts while discussing changes and contradictions between the codes. Once all codes were developed at their responding levels, we created categories and a thematic framework to explain the patterns found. The larger research team, including people with lived experiences in HIV, substance use, houselessness, and violence, reviewed the final themes for consistency and accuracy.

## 3. Results

### 3.1. Participant Characteristics

Fifty-four people were screened for participation in this study; 20 did not meet the inclusion criteria at the study start, another 2 were excluded later due to not having an HIV diagnosis, and 1 for feeling too ill to continue data collection. The final sample was 32 participants; 18 participants self-identified as men, 10 as women, 2 participants identified as Two-spirit, and 2 as non-binary or other. Sex at birth was similarly distributed among participants, 46.9% of the group were heterosexual, with 25% saying their orientation was gay, and another 25.1% responding as bi-sexual or other. Table 1 reports the sociodemographic characteristics of the 32 participants.

### 3.2. Childhood Trauma Questionnaire

Figure 2 presents the results from the Childhood Trauma Questionnaire by sex (Figure 2A) and by gender (Figure 2B). Only five persons did not experience any type of abuse. People experienced multiple types of abuse, and 70% of females and 50% of males reported severe and moderate sexual abuse. Females also experienced significant emotional abuse (60%) compared to males (38.5%).

### 3.3. Barriers

Participants reported numerous barriers to HIV care at the individual, healthcare, and social and structural levels, as seen in the statements shown in Table 2.

#### 3.3.1. Individual Factors

##### HIV Diagnosis

Most participants described their HIV diagnosis as a “death sentence”. They expressed feeling overwhelmed with shock, disbelief, confusion, and denial. Women experiencing houselessness and substance use dependence shared that their diagnosis was profoundly confusing and overwhelming. For example, participant 39 recalled feeling “confused” and “spaced out” (Table 2; Quote 1). Others felt embarrassment and internalized stigma. Men were more likely to recall feeling angry leading some to suicidal ideation and attempts. Participants explained that the combination of these emotions made it difficult to focus on the supports offered and prepare for next steps.

##### Mental Health Challenges

Many participants reported experiencing unresolved traumatic experiences beyond their HIV diagnosis. They shared the profound impact of these experiences on their mental health, which, in turn, hindered their ability to cope with and navigate their day-to-day functioning (Table 2; Quote 2). Women, men, and non-binary persons shared ongoing feelings of loneliness and hopelessness. For example, participant 46 shared how feeling hopeless led her to think “what’s the point?” in even starting HIV treatment (Table 2; Quote 3), while participant 58 experienced a loss of hope as “[losing] my mojo. I’ve lost my umph, my energies” which made him miss half of his HIV appointments. Women reported experiencing trauma related to separation from family members such as mothers and children and for some, substance use became a coping strategy to process traumatic experiences such as losing a mother (Table 2; Quote 4).

##### Substance Use Deterring HIV Care

Participants who used substances reported that their substance dependence directly interfered with their day-to-day functioning and, for some, their HIV care (Table 2; Quotes 5–7). Health was not a priority for many people who actively used substances. HIV care was affected as many reported being unable to focus on anything beyond acquiring and using their substance, leading them to forget appointments, or feeling increasingly anxious when attending medical appointments (Table 2; Quote 8).

##### COVID-19

Men and women reported the COVID-19 pandemic affected their health and mental well-being. They recounted increased feelings of isolation, depression, worry, and anxiety due to fear of acquiring COVID-19 or other illnesses with a compromised immune system (Table 2; Quote 9), and loss of income.

##### Experiences of Violence

Several participants commented on violence across their lives (e.g., intimate partner violence, sexualized). Participant 39, who was houseless and struggling with substance use at the time of diagnosis, shared a particularly harrowing story of constant sexual assaults where she was “praying that I would catch it [HIV], … I thought, if I have HIV, then there’s automatic consequences for the guys that want to do whatever to my body that I don’t agree to”. Women and non-binary participants discussed how experiences of intimate partner violence and abusive relationships impacted their HIV care. For participant 42, testing for and preventing HIV acquisition became hard as their partner would refuse to be tested for STBBIs, leaving them frustrated and alone because of the “lies and the cheating and the manipulation towards me”. Participant 46 noted that her engagement with HIV care broke down because her boyfriend was “abusive, so he keeps me tied in a lot” (Table 2; Quote 11).

#### 3.3.2. Healthcare Factors

##### Programmatic/Administrative

Participants shared some inconveniences with the delivery of primary and HIV services. Men, women, and non-binary persons discussed problems with appointment waiting times which sometimes deterred them from attending primary care clinics: “just because of the waiting period, I just decided I’m not even gonna bother getting checked out” (Table 2; Quote 12). Men and women shared they were hampered by a lack of service options for their HIV care outside “9–5 business hours”. Men remarked that the ability to obtain HIV drugs “should not be based on someone’s salary” and commented on the need for HIV services outside metropolitan areas (e.g., rural communities; Table 2; Quote 13).

##### Lack of Follow-Up Care and Support During HIV Diagnosis

As reported above, some participants felt lost and unsure of what to do after diagnosis. These feelings were exacerbated by the lack of follow-up care, education, and treatment relating to HIV from primary care providers. Participants desired more information and guidance after being diagnosed as “that was the moment when I really needed someone” and many were “expecting to feel a little empathy” as they went through emerging emotions. Women discussed the intersecting experiences of being houseless and using substances at the time of diagnosis more, leaving them even more confused during the process and overwhelmed as to where to ask for help (Table 2; Quote 14).

##### Compounded Stigma, Discrimination, and Insecurity in Health Settings

Participants reported routinely feeling stigmatized and discriminated against by healthcare providers outside of the HIV care teams, such as nurses, pharmacists, staff in hospitals, emergency rooms, and prisons. Across genders, participants described coldness, prejudice, lack of empathy or support from service providers in primary care settings, hindering their engagement in care (Table 2; Quote 15). Disappointment, frustration, and distrust were shared mainly among women who used substances because of the negative experiences they reported while seeking healthcare. These women recounted experiencing compounded stigma about their intersectional identities of HIV status, substance use, and houselessness as primary service providers would not prioritize their needs, they mocked them, or discussed their stories out loud (Table 2; Quote 16). Participant 55 remarks that she received vicious treatment not only because she had HIV but also because she was “addicted”. She explained that it is those who experience HIV and substance use dependence who are subjected to the worst treatment in emergency rooms as “they make US sit there forever. Like, just US, I’ve seen them put up through other people”. Men recounted discriminatory experiences within the criminal justice system as prison staff would disclose their HIV status without consent, leaving them angry and resentful, and discouraging them from future HIV care. Women recounted unsafe experiences in emergency and primary healthcare environments, while one man described being harassed by a security guard while trying to attend his HIV appointment.

##### Lack of Social Supports

Participants identified the reduction in HIV social support groups as another barrier to care. Participants of all genders described a time when several social groups were supporting PLHIV beyond biomedical care. While participants noted that these groups slowly disappeared before the COVID-19 pandemic, they mentioned there were even fewer after the onset of the pandemic. They described these “social gatherings” as an invaluable aspect of their maintenance in care because they offered informal mental health support for people during their journey with HIV (Table 2; Quote 17). Participants also shared disappointment about the lack of peer support for their HIV medical care, particularly during diagnosis. Having someone “that has the same thing I do” was understood as providing closer and more intimate understanding and community than medical professionals (Table 2; Quote 18).

##### COVID-19

Participants described reduced service availability (Table 2; Quote 19) during the COVID-19 pandemic, and people without a phone or internet access were particularly affected by this change. Disrupted care meant people could no longer interact in a space they relied on for social connection and support. Men and non-binary persons reported no disruptions to their biomedical care (i.e., could continue medication), yet they expressed a sense of grief in “losing this [social space] … it was our meeting place in a sense” leaving many feeling even more “segregated” during the pandemic as “this [HIV care site] is the only place that you can go to connect”. Likewise, men and women felt a loss of personal connection with their HIV service providers, as many organizations shifted their services to virtual appointments or hybrid care. While this change did not impede participants from remaining linked to medical care, it did create a loss of connection with HIV service providers.

##### Lack of Prevention Strategies

Participants shared concerns over the lack of STBBI education and prevention strategies for people who are most at risk (Table 2; Quote 20). Participants also emphasized a concern over the lack of outreach, street-based nursing, and providing care that meets people where they are at, given the number of people experiencing houselessness in Manitoba who are unable to attend traditional HIV care (Table 2; Quote 21).

##### Inaccessible Mental Health Services

Women shared stories of facing many bureaucratic hurdles to getting into substance dependence programs and a number had unsuccessful and frustrating experiences, deterring them from trying again (Table 2; Quote 24). Participant 39 described her journey of sleeping in hospital emergency rooms for six months while waiting to enter detox as “discouraging”, at times pushing her to “go back to drugs”. Women emphasized that emergency departments should aim to “[not] turn them [people with substance dependence] away. They could actually be in danger of harming themselves or others”. Men in our study were more likely to note long wait times and a limited number of mental health professionals, which frustrated participants seeking mental health supports (Table 2; Quote 25).

#### 3.3.3. Social and Structural Factors

##### Housing

Many participants did not have stable and long-term housing. In particular, for women struggling with substance use dependence, the instability associated with houselessness made it hard to focus on their HIV care, as their priorities were on “surviving out there” (Table 2; Quotes 24–26). Additionally, participants of all genders reported increased victimization, such as constant thefts of their belongings, including their HIV medications, while experiencing houselessness (Table 2; Quotes 27–28). They explained that if their medication is stolen, it can take a long time to replace it because of the steps involved in calling care providers, dealing with pharmacies, and applying for medication funding. Participants mentioned increased anxiety due to the possibility of an increased viral load, heightened infection progression, or a greater ability to transmit HIV.

##### Stigma and Discrimination

Many participants experienced enacted stigma and discrimination in their social circles because of their HIV status leaving them without adequate social circles to support their HIV care. Women and non-binary persons described feeling frustrated when people shared their HIV status without their consent and a number of participants said they felt alone, insecure, and unsure about who they can share their HIV status with those social circles without potential repercussions (Table 2; Quote 29).

##### Lack of Social and Structural Supports

Men and women shared that a lack of comprehensive social and structural supports such as financial assistance hinders their HIV care. For some participants, being unable to acquire financial assistance and an increased cost of living has forced them to find other ways to “survive” such as boosting (stealing) or fighting people (Table 2; Quote 30). Men and women also expressed a lack of affordable transportation options to attend their HIV appointments as the reason “why I miss them [appointments]”.

**Figure 3:**
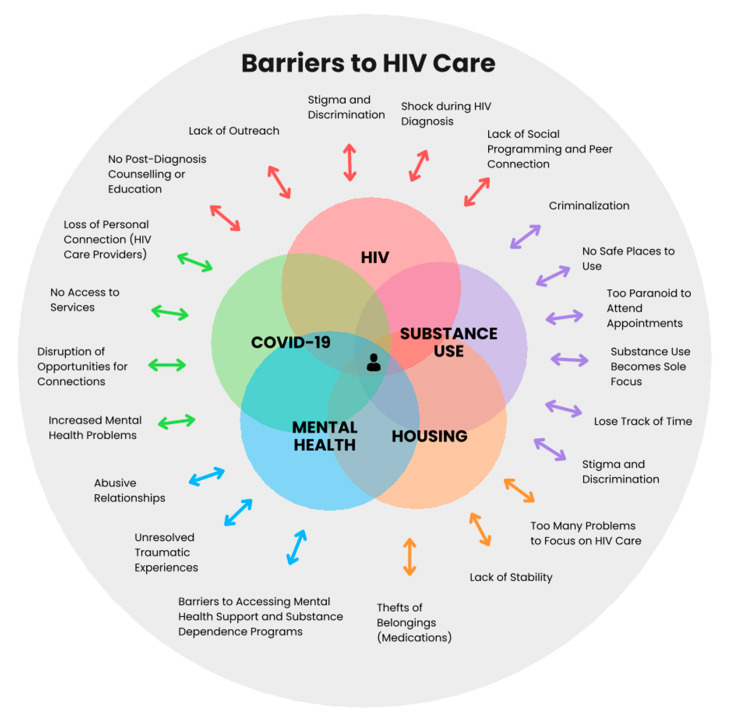
Barriers to HIV care in Manitoba. Model of understanding the interconnected barriers in HIV care experienced by people living with HIV in Manitoba.

Figure 3 provides a holistic conceptualization of the interconnected barriers experienced by PLHIV in this study.

### 3.4. Facilitators

Table 3 summarizes the most representative quotes by each level of facilitators reported by participants.

#### 3.4.1. Individual Factors

##### Stopping Substance Use

Some participants mentioned that ending substance use helped them focus on their HIV care. They described stopping substance use as a welcome change because they were better able to focus on other priorities and plan their lives, which they described as an “impossible task” when using substances (Table 3; Quote 31).

##### Will to Survive

Some participants of all genders described how having an innate will to survive is what has kept them motivated to engage with HIV care. This intrinsic motivation to stay alive was necessary for them to maintain their HIV care despite many challenges in their lives such as the trauma associated with the death of friends and family members (Table 3; Quote 32).

#### 3.4.2. Healthcare Factors

##### HIV Service Providers

At the healthcare level, the primary facilitator for all participants was the non-judgmental and above-and-beyond care they received from HIV service providers. Many participants were moved to tears when describing the importance of providers to their care and their lives. Being close with their service providers allowed participants to be “honest about what’s going on in [their] life”, without feeling judged (Table 3; Quote 33). Participants who used substances felt these relationships as “somebody cares about me”, because at times they feel that “nobody gives a shit about me, they don’t. Nobody cares, because I’m a drug user”. Participants were clear in differentiating that the care from their HIV service providers is unique and unlike the care from other medical services. Men were more appreciative of HIV service providers who involved them in designing treatment plans as they would empower them to participate in their HIV care.

##### HIV Healthcare Environments

Participants also noted the importance of welcoming HIV healthcare settings (Table 3; Quotes 34–36). Many participants shared that they felt safe at these sites because their interactions with providers were accepting and non-judgmental. Participants said staff welcomed anyone who needed help, regardless of how they presented to care, were friendly from the moment people walked through the door, remembered people’s names, and offered snacks or basic supplies. Similarly, participants felt more connected with their care when they received services for other health needs beyond their HIV care (Table 3; Quote 36).

##### Social Supports

Participants reported that incorporating social support as part of their services was beneficial for their wellbeing. Men, women, and non-binary persons emphasized social programs and opportunities for connections with peers allowed them to understand their disease better and remain hopeful about their care. They described educational workshops as empowering, especially among people newly diagnosed, as this engagement fostered a sense of community and enabled knowledge sharing among peers (Table 3; Quote 37). For many, these programs are “like the best medicine, the social part”. Similarly, other resources outside of HIV biomedical care such as help with transportation costs and using a food bank at an HIV care site motivated people to return (Table 3; Quote 38).

##### Post-HIV Diagnosis Emotional Support and Education

Participants who received emotional and educational support post-diagnosis described feeling more informed and able to connect and maintain engagement in HIV care. For women, this support created emotional safety and instilled hope to continue their lives. Men appreciated genuine service providers who did not rush to get them out of the door, thus ensuring that they “understood what was going to happen”. Men and non-binary persons found it easier to make the first connection with their HIV care when service providers were clear and organized in guiding them through the process (Table 3; Quote 39).

#### 3.4.3. Social and Structural Factors

##### Support Networks

Participants greatly emphasized the role their support networks (e.g., family, friends, partners) play in initiating and maintaining their HIV care. Support networks that are open, understanding, and non-judgmental about participants’ HIV diagnosis and care needs (e.g., reminding of medications) were described as the most important in sustaining motivation (Table 3; Quotes 40–41).

##### Structural Supports

Women who received housing and financial support were better able to focus on their HIV care. For example, participants described housing options that supported their HIV care through wrap-around services including culturally safe care, sharing appointment reminders, and arranging transportation for their medical needs. One participant’s story highlights the importance of the holistic care she received in a recovery home with wrap-around services supporting her substance use dependence, Child and Family Services journey, and HIV diagnosis (Table 3; 42).

**Figure 4:**
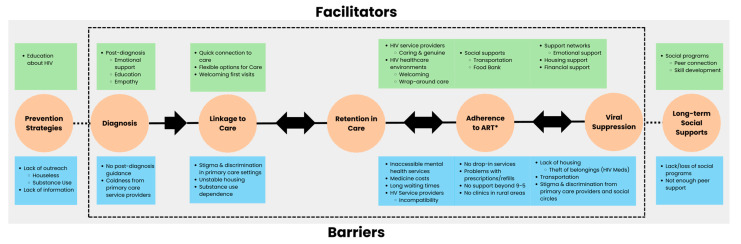
Barriers and facilitators to HIV care in Manitoba. Facilitators and barriers across an extended cascade of HIV care.

Figure 4 summarizes the barriers and facilitators in each step of an extended HIV care cascade. Participants remarked on the importance of expanding the traditional HIV cascade of care to include prevention strategies and long-term social support for a more person-centered HIV care in Manitoba.

## 4. Discussion

The experiences of women, men, and non-binary PLHIV interviewed in this study point to a complex syndemic of health and social conditions that cluster with HIV to facilitate or impede access to and maintenance of HIV care in Manitoba.

Mental health challenges including traumatic experiences (e.g., sexualized violence), problematic substance use, houselessness, stigma and discrimination, and inaccessible healthcare and social services interacted and exacerbated negative health outcomes. The findings from our study highlight how trauma and mental health challenges can affect people’s day-to-day functioning and, therefore, their HIV care. The literature suggests the negative biological interactions of these intersecting conditions. Mental health diagnoses commonly associated with experiences of trauma, such as depression and post-traumatic stress disorder, have been found to enhance inflammation and suppress antiviral immune responses potentially increasing the risk of acquiring other diseases [42,43]. Participants described receiving their HIV diagnosis as traumatic, with some men reporting suicidal ideation and attempts. Similarly, traumatic experiences, particularly those experienced during childhood, have been linked with accelerated HIV disease progression and overall negative quality of life [44]. Mental health challenges and traumatic experiences have also been found to diminish HIV treatment outcomes (e.g., viral suppression, mortality) for women [45], men and trans women [22,46], and those experiencing houselessness [24,25]. The harrowing and common experiences of interpersonal and sexualized violence among women in this study along with the increased childhood experiences of sexual and emotional abuse among females should be regarded as a concerning public health issue. Past research on the syndemic of gender-based violence, HIV, and substance use suggests that these forms of violence might increase the risk of HIV and STBBI transmission [47].

Many participants in this study resorted to substances to cope with their stressors, trauma, and lack of mental health services. Increased substance use closely interacts with trauma, mental health disorders, and HIV [34,48]. Our findings have highlighted the challenges that substance use can create for people to engage with HIV care such as being too physically unwell to attend an HIV clinic. However, it is crucial to note the biological impacts that increased substance use could have on PLHIV’s health. Approximately 71.8% of females and 43.4% of males among those with new HIV diagnoses in Manitoba reported injection substance use with methamphetamine as the substance of preference (62.4%) [4]. Substance use, including methamphetamines, can increase HIV transmission, lead to more rapid HIV progression and overall worse health outcomes for PLHIV [13,14,49].

Beyond the biological implications of the cluster of diseases described above, our findings portray how these experiences are shaped by social processes that hinder participants’ engagement with HIV care. Despite having histories of childhood trauma and mental health challenges, many participants mentioned several barriers to mental health services they needed, particularly those with substance dependence. Participants spoke positively about past peer support groups, many of which no longer exist, and reported limited peer social spaces where they could support each other’s mental health and HIV journey, interventions which have been described as cost-effective ways to improve HIV indicators [50]. These findings show a need for increased investment in psychosocial interventions to support PLHIV who have experienced trauma which can improve psychological wellbeing and health outcomes [44,51].

The COVID-19 pandemic amplified the interconnected barriers to care for PLHIV. Participants reported increased mental health challenges, loss of personal relationships with their HIV care providers, and limited opportunities to connect with peers socially. Qualitative research with First Nations PLHIV in Manitoba also found increased mental health concerns and experiences of discrimination leaving many isolated during the pandemic [52]. As healthcare systems move out of pandemic restrictions, it is important to consider how the pandemic exacerbated disparities in underserved communities [6,52,53,54,55,56] to enable reconnections to care.

The participants shared various experiences of internalized stigma, which has been linked with a lower retention in HIV care in adolescent girls and boys [26]. Our findings showed that participants experienced intersectional stigma (i.e., a compounding of stigma and experiences of discrimination based on more than one intersecting identity) [57] by primary healthcare providers and social networks because of HIV status, unstable housing, or substance use. This led to participants feeling angry and disappointed with providers who should deliver care safely and respectfully, leading to distrust and reluctance to return to places where they had experienced discrimination. Intersectional stigma has been described across HIV syndemic research as being a key factor influencing HIV transmission, mental health, substance use, and barriers to HIV care [57]. Our findings build on a growing body of work in the literature calling for more comprehensive interventions to address the HIV intersectional stigma to reduce negative health outcomes and better connect people with care [58,59]. Other studies have shown the promising results of decreasing HIV stigma and discrimination in primary healthcare settings by using targeted educational campaigns which could be explored in settings that are mandated to provide healthcare for all Manitobans [27].

Social and structural factors interacted with health conditions to hinder HIV care in this study. Two-thirds of participants were living below the official Canadian poverty line (CAD 25,471/year [60]). Poverty affects every aspect of a person’s life, and for people experiencing houselessness, the negative impact on linkage and retention in HIV care cannot be overstated [61,62]. The findings from this study show that PLHIV who are experiencing houselessness have other competing priorities before HIV care. Houselessness and poverty led many participants to find ways to support themselves, such as stealing, which placed them at higher risk of criminalization and victimization. Likewise, theft of belongings made it hard for people experiencing houselessness to adhere to their medication and treatment, since besides “trying to survive”, they must spend time obtaining new medications. These participants felt worried and anxious about their viral load increasing to a transmissible level due to missed doses. These worries are not unfounded as at an individual level non-adherence to antiretroviral therapy can lead to increased HIV replication [63] and reduced CD4 cell counts [64], and at a population level it can lead to higher rates of HIV transmission, healthcare utilization [65,66] and increased mortality [67,68].

According to participants, current responses to these problems are insufficient as they remarked on a lack of preventative strategies, such as outreach and street-based nursing for PLHIV experiencing houselessness, that could engage people at risk. Similarly, participants shared frustration over the lack of universal coverage of HIV medicine in Manitoba (approved in 2024, after completion of this study) as some people without coverage cannot afford to pay their annual Pharmacare deductible, adding the financial consideration of cost of managing comorbidities to the choice of HIV care. We call on decision makers to prioritize evidence-based [62], accessible, permanent, and emergency housing options for PLHIV, which would support increased access to and retention in HIV care [69].

The findings in this study also suggest facilitators that enable participants’ engagement in HIV care, such as caring service providers and welcoming healthcare environments. The emotion with which many participants described their HIV care providers, and a welcoming environment highlights the enormous work providers have done to create trust and safety. For many participants, HIV clinics were among the few safe spaces in their lives. These experiences align with past qualitative research that has emphasized how providers who embody care, compassion, empathy, and empowerment in their patient interactions serve as facilitators in engaging PLHIV with care [25,70,71,72,73,74,75,76]. Funding to retain and support these services is critical, given the body of evidence on the importance of these patient interactions in facilitating people into HIV care. 

The main limitations of this study include the required time commitment for data collection sessions. Not all PLHIV in Manitoba may have had time to commit to this study, and we may have missed some perspectives. However, our research design allowed for low-barrier and flexible data collection methods to include participants experiencing complex situations, providing confidence that our findings represent diverse populations. Additionally, we may have missed PLHIV in communities outside Winnipeg and Brandon who have different barriers to their care. To address this, we engaged with community partner organizations, enabling the participation of PLHIV who have received care while living outside of the two largest cities in Manitoba. An additional limitation is that we did not measure the biological factors that may serve the basis of social–biological interaction. This is a complex task that was beyond the scope of the current project.

## 5. Conclusions

Manitoba is the province with the second-highest number of new HIV diagnoses in Canada. Women, men, and non-binary people face barriers to HIV care including individual, healthcare, and social and structural factors intersecting and challenging the achievement of UNAIDS 95-95-95 goals [1]. Using syndemic theory, our findings add to the literature on how specific biological (e.g., substance use, mental health) and socioeconomic (e.g., poverty, social seclusion) create adverse effects in PLHIV’s engagement with HIV care [11,33]. These findings shed light on the interacting diseases and inequalities that further marginalize the health of underserved populations. In addition, our findings emphasize the need to move beyond siloed interventions and, instead, focus on holistic person-centered care options that address the interconnected root causes such as poverty, houselessness, and stigma and discrimination contributing to the rising HIV diagnoses in Manitoba. While managing all the conditions PLHIV are experiencing (Figure 3) might seem organizationally complex, there are integrated interventions (i.e., addressing multiple problems) such as offering education for HIV, individualized and group mental health supports, housing assistance, supervised injection programs within HIV and primary care facilities, and collaborations between academic, civic, and community organizations, that show encouraging results in contexts similar to Manitoba [69,77,78,79]. Future research should assess the feasibility of implementing such interventions as part of the HIV care model in Manitoba and the effects such interventions have on immune activation as a surrogate measure of converging risks.

## Figures and Tables

**Table 1:** Sociodemographic characteristics of participants in the qualitative interviews.

Variable (N: Participants Who Answered)	Frequency n (%)
Age in years (N = 32)	44.03 years (24–63)
Mean (Range)	
Gender Identity (N = 32)	
Woman	10 (31.3)
Man	18 (56.3)
Trans Woman	0
Trans Man	0
Non-Binary	1 (3.1)
Two-Spirit	2 (6.3)
Other	1 (3.1)
Prefer not to say	0
Sex (N = 32)	
Male	21 (65.6)
Female	10 (31.3)
Intersex	0
Prefer not to say	1 (3.1)
Sexual Orientation (N = 32)	
Lesbian	0
Gay	8 (25)
Bisexual	6 (18.8)
Asexual	0
Heterosexual	15 (46.9)
Pansexual	0
Other	2 (6.3)
Prefer not to say	1 (3.1)
Cultural Background (N = 32)	
Indigenous–First Nations	15 (46.9)
Indigenous–Métis	4 (12.5)
White/European	4 (12.5)
Southeast Asian	4 (12.5)
Other	5 (15.6)
Marital Status (N = 27)	
Single	18 (56.3)
Married	0
Divorced	2 (6.3)
Common Law	7 (21.9)
Widowed	0
Other	0
Highest Level of Education (N = 30)	
K-12	21 (65.6)
Certificate, Diploma, vocational course from an	6 (18.8)
educational institution	
Bachelor’s Degree	3 (9.4)
Master’s Degree	0
Doctorate	0
Other	0
Income (N = 31)	
<10,000 CAD/Year	12 (37.5)
10,000–19,999 CAD/Year	8 (25)
20,000–29,999 CAD/Year	2 (6.3)
30,000–39,999 CAD/Year	3 (9.4)
40,000–49,999 CAD/Year	3 (9.4)
>50,000 CAD/Year	1 (3.1)
Prefer not to say	2 (6.3)
Housing Situation *	
Living Alone (N = 32)	10 (31.25)
Living with Partner (N = 32)	6 (18.75)
Living with Children (N = 32)	3 (9.38)
Living with Roommates (N = 32)	5 (15.63)
Living with Extended Family (N = 31)	8 (25)
Experiencing Housing Instability (insecure housing, shelter, transitional housing, houseless) (N = 32)	14 (43.75)

* Non-mutually exclusive categories.

**Table 2:** Representative quotes on the barriers experienced by men, women, and non-binary people living with HIV in Manitoba by individual, healthcare, and social and structural factors.

Category	Quote #	Quote	Participant Age and Gender
Individual Factors			
HIV Diagnosis	1	I was so in the clouds about it. I don’t even remember them talking to me about any support or anything … I do remember … I felt like garbage and like, as if nobody wanted to be around me, or I felt gross and felt really suicidal. It really embarrassed me to even talk about it.	Participant 39 Woman
Mental Health Challenges	2	I have flashbacks, and like, it interferes with my work in school, because then I’m spacing out, and it has to do with me not talking about it [sexual abuse] either because I think that if I just keep myself busy, that’ll distract whatever is going on in my head.	Participant 39 Woman
	3	I don’t know what to do with myself, and I just feel if I do anything … (soft cry), I’m just scared to do anything. I’m scared to reach out and start doing things with my life because I always end up backwards headwind, and it just goes away. So, I just think what’s the point? What’s the point in trying to do the same thing? … It’s just recently I’ve been wanting to [get back on HIV treatment] because I just felt like there was no point in trying to fight this disease because there wasn’t much to live for.	Participant 46 Woman
	4	It was really hard to lose your best friend [mother] and someone who was, your rock, you know. I just turned into an adult [and] I was grieving. I didn’t understand grief and loss. I didn’t realize that there’s a cycle and you can actually deal with it without turning to drugs and alcohol because … I missed my mom, I needed my mother.	Participant 40 Woman
Substance Use Deterring HIV Care	5	When they say it’s [having substance use disorder] like, a flu, or whatever. It’s kind of like a flu and it’s not because it is definitely worse. Like you can’t even move. You can’t get up to feed yourself or you can’t get up to even bathe yourself or anything.	Participant 43 Woman
	6	I wasn’t consistent [with HIV medications], you know, like you gotta take it every day. And I wasn’t, you know, it’d be going off and be like, I didn’t go a week without it, but maybe three days. Sometimes it was like, ‘Oh, my God, what am I doing?’ but I was using [methamphetamine] a lot. So, when you using too and you don’t care, your health, like your health doesn’t really matter.	Participant 64 Man
	7	I just have addictions, like, you know, a lot of my days are based around what I do get like [high].	Participant 55 Woman
	8	Like, going to get into an [medical] appointment? Feeling the social anxiety oh no, no. Oh, they think I’m high or they know I’m high or whatever.	Participant 39 Woman
COVID-19	9	Healthcare settings, there was a fear of going to … because I didn’t want to go in because even though my CD4, is okay, I still have this fear of getting in, getting sick.	Participant 33 Man
Experiences of violence	10	I feel pretty drained I guess tired. I really wanted to get on my [HIV] medication, but it’s just like, with my boyfriend around, I could never get to go do what I need to do … I connected with them [HIV care] once my boyfriend went into jail … but before that I didn’t see them for like two years.	Participant 46 Woman
Internalized Stigma	11	I am ashamed of myself. I could say that. I would say to myself I am no longer clean I am dirty and contaminated with the virus.	Participant 35 Man
Healthcare Factors			
Programmatic/Administrative	12	The only thing is sometimes the waiting … It could take a week, two weeks, or three months to get an appointment for something that you might want to be able to see someone like that day.	Participant 57 Two-Spirit
	13	Have an HIV clinic on the reserve, or more services for HIV in the reserve … I was flying in and out to get here for appointments… It would be expensive because we would be using money everyday here, and we had to bring the kids over here.	Participant 49 Man
Lack of follow-up care and supports during HIV diagnosis	14	Because I was homeless at the time, so I had a lot that I was missing to actually be focusing on one thing [HIV diagnosis] so that’s why my head was like everywhere … I think I was looking for someone to tell me that everything was going to be okay and that what I needed to do was do these steps to-to get where I’m trying to go and not give up. I honestly can’t remember when they told me I had HIV. I’m pretty sure they just gave me a phone number and where to call and if I needed support and someone to talk to and that was it.	Participant 39 Woman
Stigma, Discrimination, and Insecurity in Health Settings	15	They [prison staff] come in and they’re like ‘hey Mr. you know you [got] AIDS right? Do you know you have AIDS right?’ I thought he was joking or whatever but then he is not… A little bit of support a little bit of sympathy like, buddy, you just told me I have fucking AIDS and you just come here like nothing like ‘Hey you know you got AIDS right’. How much of a slap in the face do you think that is? I was a kid man, 23 years old.	Participant 52 Man
	16	The hospitals are fucking ridiculous. I can’t go to emergency rooms, and some of those nurses are vicious because they’re judgmental. You hear them talking because I got HIV, they treat us different because we’re addicted. They treat us differently just because we have addictions … Those chicks [nurses] are mean I hate going to emergency rooms now because a lot of my other friends said the same thing before, they won’t go to hospitals and like the emergency rooms because they treat us so shitty there.	Participant 55 Woman
Lack of Social Supports	17	The environment here [hospital] is very sterile sometimes … Well, we started it [food bank] and we had coffee out, and people would sit down, and they were talking, and you would network. I had more confidence in not thinking that I was going to die anytime I had the flu, or anytime I had a spot. Because I met people at the food banks, while I was volunteering, that were there for like 10 years, 20 years, 30 years, you know, I met people there and we all sat around and we talked and we got to know each other. And if somebody passed away, we knew they were dead. Now. You [don’t] hear about it? Maybe on Facebook.	Participant 54 Woman
	18	Definitely someone who’s got it [HIV], someone’s got already. A nurse and doctor no-no, someone who’s got already … More believable, yes. I mean I believe doctors, but you know they still got their own jargon … I want to hear it from someone who’s gone through it.	Participant 58 Man
COVID-19	19	Because there was places that weren’t even open. And you had to have a phone, you had to have internet.	Participant 39 Woman
Lack of Prevention Strategies	20	I rarely ever see, tables for resources [in shelters] … Like, this [educational materials] wasn’t there when I was on the streets and stuff. They [shelters] kind of just had a place for people to come in eat, you know, sit around and chat. There was no resources being handed out. There was posters hung up. But, I like I would change the fact that people get greeted at the door with people with a whole table of pamphlets like this. ‘Are you struggling with addiction? This is what you can do to help. And this is what you can do to get there’.	Participant 40 Woman
	21	You got to have more outreach going to these people [experiencing houselessness] and saying, ‘Hey how are you guys doing?’ I’ve never seen that here in Winnipeg. Out of all the people in like back lanes and stuff I’ve never seen anybody talk to him like a human being. ‘How are you doing? Are you okay? Is everything okay with you? Sure. Do you need anything? No. Just checking in to make sure because you’re a human being’. … They don’t outreach you gotta reach out to them.	Participant 52 Man
Inaccessible Mental Health Services	22	I used to have a hard time getting into detox, I would spend nights at hospitals in the waiting room trying to get into [a] detox place. ‘What am I doing wrong?’ I’m saying that I’m trying to get out of this, and they seem to not be taking me serious or something, or seem to think I could do it by myself … It was discouraging for me to speak up for myself. Yeah, like different hospitals. Sometimes, I’ll just give up and just go back to drugs.	Participant 39 Woman
	23	If I want to talk to a psychologist and fuck it’s two years … And then suddenly you get a psychologist. He’s like, yeah, you don’t need me because you’re gay or because you’re this.	Participant 51 Man
Social and Structural Factors			
Housing	24	Because I was homeless at the time so that’s why my head was like everywhere … Well I don’t have a phone, I don’t know where to go, you know, I’m homeless, struggling with an addiction and you’re just gonna set me off on the street expect me to figure out things by myself.	Participant 39 Woman
	25	I had a rooming house experience, but I left it. Fucking awful, overrun with mice. Addictions and homelessness, just like confusion … I’ve been trying to get a place which is a lot. Yeah, I just was trying to survive out there.	Participant 55 Woman
	26	Having a place to go at night. It’s called mine. Not stay out [at] somebody else’s place. … Like people who are homeless and HIV and all like it’s really important for them to be in stabilized situation to where they don’t get sick and their immune system is like not sick.	Participant 41 Woman
	27	They [HIV doctors] only give me a week’s worth [of medication] at a time because I live on the streets, and bags get stolen a lot so, they don’t want me to have all my meds on me and when I fall asleep and my bag goes missing … Having the stability of my own place having a steady routine that’s how you get into not forgetting and take your pill every day. Because you have a home you go home that you can fell sleep.	Participant 42 Non-Binary
	28	When my pills got stolen, they were in my room. I was in this rooming house, they were in a bag, my bag that I take with me everywhere. And they got stolen. And I wasn’t able to go on my meds for two weeks, because they were only given to you once a month. Tried coming here [HIV health centre]. Come here, I couldn’t see the doctor, of course.	Participant 59 Man
Stigma and Discrimination	29	After that [HIV diagnosis], everyone started treating me different. You know, everyone wanted to stay away, like, didn’t want to shake my hand and didn’t want to be around me. It was just really hard.	Participant 46 Woman
Lack of Structural Supports	30	Zero [money] it’s hard for fucking four months already and got no support, no money, no fucking nothing … I try to fucking walk around and I get paid just fucking beating somebody in streets	Participant 32 Other

**Table 3:** Participants’ quotes regarding facilitators to HIV care, by individual, healthcare, and social and structural factors.

Category	Quote #	Quote	Participant Age and Gender
Individual Factors			
Stopping Substance Use	31	Things are starting to get a lot better because I have stopped doing drugs like I used to inject. So, I quit like eight weeks ago now. So, things are getting a lot better for me.	Participant 46 Woman
Will to Survive	32	You have to learn how to have a thick skin. Take things with salt. You sort of have to look at it. If I was in that situation, what would I do? To help people and stuff like that.	Participant 48 Man
Healthcare Factors			
HIV Service Providers	33	I’m very close with my nurse, my doctor. So, I’m close with a lot of the receptionist. A lot of them know me by name. And we laugh and we joke and it’s just-it’s just like I said, just having an extended support circle of family, like I wouldn’t even call them friends, they’re more like family because they know my story because they’ve known me for so long.	Participant 40 Woman
HIV Healthcare Environments	34	Only here [HIV care site] is where I got help. If I wasn’t for this place, I would probably be dead right now. I really don’t know where I would be because I have nobody for talking.	Participant 58 Man
	35	It’s just an accepting, loving environment … like it’s-it’s very friendly and welcoming, there’s no judgment here. They understand everything, you know, it’s a lot more [welcoming] than others [healthcare settings].	Participant 40 Woman
	36	I think it’s great because they’re more concerned about me as a whole, not just one little piece of me, and that one little piece of me [HIV] is really almost, I know this might not sound right., but it’s okay. It’s almost like that’s been put on the backburner, you know, they look at the results. You don’t see anything that scares them … It’s like, you know, ‘how you’re feeling otherwise, how’s your aches and pains falls?’	Participant 44 Man
Social Supports	37	How they had their little workshops and programs. I really liked one of their programs that I did … I came to all 10-10 classes … It told me a lot about my disease, and plus I got to meet people that had it too. And I really liked that too, because I felt so alone. When I first got it, I just thought I’m the only one out there [with HIV].	Participant 46 Woman
	38	And the only reason I came here was because I’d run out of food at home, and I was hungry. And I had this piece of paper from the hospital saying, I was entitled to a food bank here. I was like what the hell, I’ll go down and see if I can find out.	Participant 44 Man
Post-HIV Diagnosis Emotional Support and Education	39	They made me feel really [safe] … I was broke down crying. They were talking to me and telling me ‘it’s okay, this isn’t, you know, the end of it’ … she’s like, ‘it’s okay, there’s treatment and stuff’. Because when you hear HIV, people are like, ‘Oh, my God, I’m dying’, right? So, it’s like, that’s initially what I thought … then they-they pretty much calmed me down, made me feel safe, and eventually they were able to tell me that this is, you know, I have an appointment and stuff so and they were willing to pick me up and take me to my appointment.	Participant 40 Woman
Social and Structural Factors			
Support Networks	40	I told my cousin’s wife first. Of course, and I broke down, and she came over to hug me. ‘You are just sick, that’s all. Don’t worry there are lots of treatments out there available, you’re still young’. And then I told my sister there, and she’s like-she’s like, ‘I don’t care, you’re still my brother. Just take your meds.’	Participant 49 Man
	41	For myself, I didn’t like being looked at weird, you know, I know my mom never looks at me any different like she’s always there for me. You know so I wish more people were like my mom and just loved us unconditionally.	Participant 62 Woman
Structural Supports	42	So, I got in contact with people there [housing services] because I was homeless, I was struggling to stay sober… But when I got my own place [without wrap-around supports], I just went off the handle …My first month’s rent money went to alcohol, drugs, more alcohol, it was-it was crazy … So, and then I got addicted to meth like right before I was kicked out … I’ve been there since the program opened [new recovery home with wrap around services]… I really have, where I live is a support. Like I have an unending circle of support … So, when I get the [HIV care] appointments, I-I write them down, and then make sure to put it in my calendar. And I made sure to tell them so that they know ahead of time. They’re like, ‘does she need a ride?’	Participant 40 Woman

## Data Availability

Individual qualitative participant data will not be available since it contains potential and sensible identifiable information. Data dictionaries, qualitative codebooks, and quantitative data will be available upon request on a case-by-case basis.

## Supplementary Materials

The following supporting information can be downloaded at: https://www.mdpi.com/article/10.3390/tropicalmed9120287/s1, Supplementary File S1: Operational Definitions; Supplementary File S2: Participant Survey: Demographics & Life Circumstances. References [80,81,82,83,84,85] are cited in the Supplementary Materials.
